# The liver proteome in a mouse model for *Ascaris suum* resistance and susceptibility: evidence for an altered innate immune response

**DOI:** 10.1186/s13071-019-3655-9

**Published:** 2019-08-14

**Authors:** Gwendoline Deslyper, Celia V. Holland, Thomas J. Colgan, James C. Carolan

**Affiliations:** 1 Department of Zoology, School of Natural Sciences, Trinity College Dublin, Dublin 2, Ireland; 2 School of Biological, Earth and Environmental Sciences, University College Cork, Cork, Ireland; 3 Department of Biology, Maynooth University, Maynooth, Co. Kildare Ireland

**Keywords:** *Ascaris*, Liver, Proteome, Shotgun mass spectrometry, Innate immune response, Mitochondria, Retinol

## Abstract

**Background:**

Ascariasis is a neglected tropical disease that affects 800 million people worldwide. Whereas most people only experience light worm burden, some people experience heavy worm burdens even after several rounds of chemotherapy, a phenomenon known as predisposition. Such heavy infections are associated with more severe symptoms and increased chronic morbidity.

**Methods:**

In order to investigate potential mechanisms that may explain the observed predisposition, we infected mice with the porcine ascarid *Ascaris suum* using an established mouse model with two different mouse strains, where the C57BL/6J strain is more susceptible to infection and therefore a model for heavy infection and the CBA/Ca strain is more resistant and thus a model for light infection. At day 7 post-infection we investigated the liver proteome, using shotgun mass spectrometry, of both infected and control mice of each strain.

**Results:**

We identified intrinsic differences, between the two mouse strains, in both oxidative phosphorylation proteins and proteins involved in retinol metabolism. Additionally, we found differences between the two mouse strains in activation of the complement system, where the CBA/Ca strain has higher protein abundances for lectin pathway proteins and the C57BL/6J strain has higher protein abundances for complement inhibiting proteins. The CBA/Ca strain had a higher abundance of proteins involved in the activation of the complement cascade *via* the lectin pathway. In contrast, the C57BL/6J strain demonstrated a higher abundance of proteins involved in arresting the complement pathway.

**Conclusions:**

We observed clear differences between the two mouse strains both intrinsically and under infection.

**Electronic supplementary material:**

The online version of this article (10.1186/s13071-019-3655-9) contains supplementary material, which is available to authorized users.

## Background

The neglected tropical disease ascariasis, caused by the parasitic nematode *Ascaris lumbricoides*, infects an estimated 800 million people worldwide [1]. The disease is widespread in Africa, Asia and South America [2] and is particularly prevalent in children between the age of 5 and 12 who suffer the majority of the worm burden [2, 3]. Ascarids are also of agricultural significance as pigs are infected with *Ascaris suum*, which has a profound economic impact due to increased feed to gain ratio and liver condemnation [4].

Symptoms of ascariasis can be divided into acute and chronic [5]. Acute symptoms, though rare, can be severe and include intestinal obstruction and a characteristic allergic response, due to larvae migrating through the lungs, called Loëffler syndrome [6–8]. Chronic symptoms are more common and although these are less severe, they have a significant impact at the population level. These include malnutrition, with associated stunting, and decreased cognitive development [9].

A small proportion of the population become heavily infected and are termed ‘wormy people’, whereas the majority of the people only experience a light infection [10]. This phenomenon is termed aggregation and is a key epidemiological pattern observed in macroparasites including *Ascaris* infection [11–13]. Increased worm burden is associated with more severe symptomatology and can lead in extreme cases, especially in young children, to intestinal obstruction and even death [9, 14, 15]. Additionally, certain individuals were found to regain a similar worm burden upon reinfection, a phenomenon known as predisposition [15]. Identifying the factors responsible for predisposition and aggregation may enable the development of new therapies that will halt the parasite during its larval migration and before it causes extensive tissue damage to the host.

In order to examine the factors responsible for predisposition, a better understanding of the host’s response to the migrating larvae is necessary. Due to limitations with epidemiological studies, where it is impossible to study a large part of the parasite’s life-cycle in its natural host, it is necessary to use alternative model organisms [16]. Although pigs are natural hosts of *A. suum*, their husbandry, cost, size and lack of inbred strains makes them challenging to use under experimental conditions [16]. Other model organisms, such as mice, rats, guinea pigs, gerbils, rabbits, goats and cows are abnormal hosts, meaning that the parasite has an incomplete life-cycle [16]. In mice, the parasite follows a similar migratory pathway as in humans and pigs, making mice suitable as a model organism for early *Ascaris* infection [17]. Lewis et al. [18] developed a mouse model to explore *Ascaris* aggregation. They identified the C57BL/6J mouse strain as a model for relative susceptibility and the CBA/Ca strain for relative resistance. The difference in larval burden between the two strains was found to occur at the hepatic/post-hepatic stage of the migratory path [18].

Using this mouse model, we previously used label-free quantitative mass spectrometry to investigate the liver proteome between the two mouse strains with and without *A. suum* infection at day 4 post-infection [19]. This study revealed an inherent difference between the two mouse strains in mitochondrial proteins, more precisely for proteins involved in the oxidative phosphorylation (OXPHOS), with the CBA/Ca mouse strain having a higher abundance of these proteins than the C57BL/6J strain both with and without infection. Furthermore, a decrease in abundance of ribosomal proteins was observed under infection for both strains, when compared to their respective controls.

In the present study, we build upon our previous data and examine the livers of CBA/Ca and C57BL/6J mice with and without *A. suum* infection at day 7 post-infection (p.i.), using high throughput quantitative mass spectrometry. At this time point, the majority of larvae have migrated from the liver to the lungs, with a peak larval burden observed in the lungs for both strains [20]. Our original study at day 4 p.i. illuminated the potential molecular determinants of resistance and susceptibility between the two mouse strains and a more restricted immune response than expected. It is anticipated that by examining the response in the livers at day 7 a stronger immune signature will be apparent and provide an enhanced view of the overall response to the sustained presence of *Ascaris*.

## Methods

### Sample collection

Four mice of both C57BL/6JOlaHsd (Comparative Medicine Unit, Trinity College Dublin, Dublin, Ireland) and CBA/Ca (Harlan Laboratories, Blackthorn, UK) strains were infected with 1000 embryonated *A. suum* ova (kindly supplied by Peter Nejsum, Faculty of Health and Medical Sciences, The University of Copenhagen, Denmark). The animals were euthanized using cervical dislocation at day 7 p.i. and the livers of these mice and their matching controls were extracted and each lobe was snap frozen in liquid nitrogen separately and stored at − 80 °C. Larval counts on lung tissue were performed on day 7 p.i. on five mice of both strains in order to confirm the difference in larval burden between the strains using the modified Baermann technique [21]. The larval pellet was suspended in 5 ml of 0.9% v/v saline and 6% v/v formalin. The solutions were agitated prior to larval count in order to obtain a homogenous larval distribution in the samples and a 2 ml aliquot was applied to a nematode counting slide (Chalex Corporation, Park City, UT, USA) [20] and the number of larvae was estimated.

### Sample preparation for mass spectrometry

The left lobes of day 7 p.i. livers were homogenized in lysis buffer (LB) comprising 6M urea and 2M thiourea, supplemented with a protease inhibitor cocktail (Complete Mini, Roche, Citywest, Ireland). In order to remove cellular debris, the samples were centrifuged for 5 min at 10,000× *g* to pellet any cellular debris. Five hundred microliters of this lysate was added to 500 µl of LB and quantified using the Qubit System (Invitrogen, Dun Laoghaire, Ireland) following the manufacturer’s instructions. Seventy-five micrograms of total protein was removed and purified using a 2D Clean Up Kit (GE Healthcare, Belfast, UK) following the manufacturer’s instructions. The resulting pellet was resuspended in 50 µl of resuspension buffer (6M urea, 2M thiourea, 0.1 MTris-HCl, pH 8.0), requantified and 20 µg was removed for in solution trypsin digest. One hundred and five microliters of 50 mM ammonium bicarbonate and 1 µl of DL-dithiothreitol was added to each protein sample and the resulting mixture was incubated for 20 min at 56 °C to reduce cysteine disulphide bridges. Cysteines were alkylated by adding 2.7 µl of iodoacetamide (0.55 M) and incubated at room temperature (20–25 °C) for 15 min in the dark. One microliter of both 1% (w/v) Proteasemax (Promega, Kilkenny, Ireland) and 0.5 µg/µl trypsin (Promega) were added and the samples were incubated overnight at 37 °C. The next day the samples were briefly spun and acidified with 1 µl of trifluoroacetic acid for 5 min at room temperature (20–25 °C). Samples were centrifuged at 13,000× *rcf* for 10 min and the supernatant, containing the peptides, was removed and purified using C18 Spin Columns (Pierce, Thermo Fisher Scientific, Dublin, Ireland) following the manufacturer’s instructions. Peptides were lyophilised using a Speedyvac (Savant DNA120, Thermo Fisher Scientific) for 2 h at 39 °C and stored at 4 °C. On the day of mass spectrometry, the peptides were resuspended in loading buffer for QExactive (2% acetonitrile, 0.05% trifluoroacetic acid), sonicated for 2 min and centrifuged for 5 min at 15,460× *g*. The remaining supernatant was used for mass spectrometry analysis.

### Mass spectrometry

One microgram of tryptic peptides was loaded onto a QExactive (Thermo Fisher Scientific) high-resolution accurate mass spectrometer, connected to a Dionex Ultimate 3000 (RSLCnano, Thermo Fisher Scientific) chromatography system. A 50 cm column was used to separate the proteins using a 4 to 40% acetonitrile gradient with a 130 min reverse-phase gradient at a 250 nl/min flow rate. Data was collected using the automatic data dependent switching mode. A full MS scan was set at a resolution of 70,000 with a scan range of 400–1600 m/z, selecting the 15 most intense ions. Subsequently, an MS/MS scan was performed, with a resolution of 17,500 and a range of 200–2000 m/z.

MaxQuant v.1.5.6.5 (http://www.maxquant.org) was used to perform protein identification and LFQ normalisation on the MS/MS data following [22]. The MS/MS data were searched against both the *Mus musculus* SWISS-PROT database database (accessed July 2017, containing 16,716 sequences; 9,390,804 residues) [23] and a contaminant sequence set supplied by MaxQuant, using the Andromeda algorithm [24]. The search was performed using the first search peptide tolerance of 20 ppm, second search peptide tolerance of 4.5 ppm with cysteine carbamidomethylation as a fixed modification and N-acetylation of protein and oxidation of methionine as variable modifications. A maximum of two missed cleavage sites was allowed. The false discovery rate (FDR) was set at 1% for both peptides and proteins. The FDR was estimated using searches against a target-decoy database. Using the MaxLFQ algorithm [25], LFQ intensities were calculated from razor and unique peptides with a minimum ratio count of two peptides across samples. A minimum length of seven amino acids was needed in order to be considered for identification. Additionally, proteins were only considered for identification if more than one unique peptide for each protein was found. The MS proteomics data and MaxQuant search output files have been deposited to the ProteomeXchange Consortium [26] *via* the PRIDE partner repository with the dataset identifier PXD014508.

### Data analysis

Normalised protein intensities (LFQ intensities) were used for the subsequent quantitative analysis in Perseus v.1.6.0.7. Proteins were filtered for contaminants: only identified by site, reverse and potential contaminants. The LFQ intensities were log_2_ transformed [27] and individual replicates were allocated their respective sample groups (CBA infected, CBA control, C57 infected and C57 control) prior to protein annotation. Only proteins found in all four replicates of at least one group were retained for analysis. Subsequently, a data-imputation was performed [28] to replace any missing values. The imputated values are chosen to simulate values of low abundant proteins and are chosen randomly from a distribution using a downshift of 1.8 times and a width of 0.3 times the mean standard deviation (SD) of all measured values.

Using these imputated data, two sample t-tests and volcano plots were generated for the relevant comparisons using the standard settings of FDR cut-off at 0.05 and S_0_ = 0.1. The statistically significant differentially abundant (SSDA) obtained from these t-tests were used to generate volcano plots. Using the ‘categories’ function in Perseus, different pathways and processes could be visualised on these volcano plots which could indicate trends. A principal components analysis (PCA) was completed on normalised intensity values. Hierarchical clustering, with Euclidean distance and complete linkage, was performed using Z-score normalised intensity values.

All proteins were annotated using the UniProt gene ID. Terms for Kyoto Encyclopaedia of Genes and Genomes (KEGG) name, KEGG pathway, Interpro and protein family (pfam) were extracted for each protein. Enrichment for these terms was performed on hierarchical clustering using the Fisher’s exact test with a Benjamini–Hochberg (Ben–Ho) corrected FDR of 2%. FASTA files of all FDR positive proteins were made using Bioedit (http://www.mbio.ncsu.edu/BioEdit/bioedit.html) [29]. Protein network analysis was completed using the online tool: Search Tool for Retrieval of Interacting Genes/Proteins (STRING) [30] v.10.5 (http://string-db.org), with a high confidence setting (0.5–0.7). KEGG pathway analysis was performed using BlastKOALA [31, 32] (http://www.kegg.jp/blastkoala/). FASTA sequences were uploaded with taxonomy identified as animals and family eukaryotes and the enriched pathways were identified.

### UniProt accession numbers for proteins mentioned in text

25-hydroxycholesterol 7-alpha-hydroxylase: Q60991; 40S ribosomal protein S30: P62862; acetoacetyl-CoA synthetase: Q9D2R0; acyl-CoA desaturase 1: P13516; aldehyde oxidase 3: G3X982; all-trans-retinol 13,14-reductase: Q64FW2; C4b-binding protein: P08607; complement C3: P01027; complement C4-B: P01029; complement component C8 alpha chain: Q8K182; complement component C8 beta chain: Q8BH35; complement component C8 gamma chain: Q8VCG4; complement component C9: P06683; complement factor B: P04186; complement factor H: P06909; complement factor i: Q61129; cytochrome P450 3A13: Q64464; cytochrome P450 3A13: Q64464; cytochrome P450 4A10: O88833; cytochrome P450 4A14: Q62264; eosinophil granule major basic protein: Q61878; fibrinogen alpha chain: E9PV24; fibrinogen beta chain: Q8K0E8; fibrinogen gamma chain: Q8VCM7; haptoglobin: Q61646; haemoglobin subunit beta-2: P02089; major urinary protein 1: P11588; major urinary protein 17: B5X0G2; major urinary protein 2: P11589; major urinary protein 20: Q5FW60; major urinary protein 3: P04939; major urinary protein 6: P02762; mannose-binding protein A: P39039; mannose-binding protein C: P41317; plasminogen: P20918; platelet glycoprotein 4: Q08857; protein S100-A1: P56565; protein S100-A10: P08207; protein S100-A8: P27005; protein S100-A9: P31725; putative hydroxypyruvate isomerase: Q8R1F5; S-methylmethionine-homocysteine S-methyltransferase BHMT2: Q91WS4; tapasin: Q9R233; thyroid hormone-inducible hepatic protein; tyrosine-protein phosphatase non-receptor type 6: P29351

## Results

The mean larval burden in the lungs of the C57BL/6J mice on day 7 post-infection (*n* = 5) was 188 ± 78.7 (mean ± SD) and the mean larval burden for CBA/Ca mice (*n* = 5) was 12 ± 8.5 confirming the susceptible and resistant phenotype in the mice used in our experiment.

**Fig. 1:**
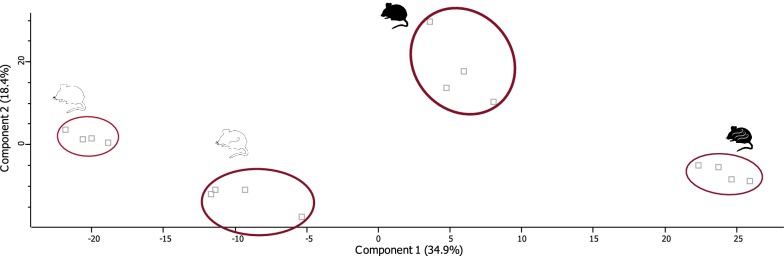
Principal components analysis (PCA) of the proteome of the left liver lobes of CBA/Ca and C57BL/6J infected with *A. suum* at 7 days post-infection. All four groups are clearly distinct from each other. The groups from left to right are: CBA/Ca control, CBA/Ca infected, C57BL/6J control, C57BL/6J infected. CBA/Ca mice are represented by the white mouse symbol and C57BL/6J mice are represented by the black mouse symbol. When infected, there are *Ascaris* larvae drawn in the mice

A PCA was performed on all proteins (Fig. 1), resolving a clear difference between the four groups and highlighting an intrinsic difference between the two strains. A PCA reduces the information of the initial variables into new variables, or principal components. These principal components are linear combinations of the initial variables and take the total variance of the data into account. This allows visualisation of a multivariate table, which is reduced to a few principal components without losing too much information from the initial variables. The PCA shows a clear difference between all four groups with no overlap. The first two principal components (PC) explain 53.3% of the total variation. PC1 (34.9%) explains most of the variation, clearly dividing the groups into their respective clusters. PC2 (18.4%) mainly explains the variation between control and infected samples. This difference is clear when comparing control samples of both mouse strains as well as for infected samples. Most evident is the clustering per mouse strain, with each strain having the clusters of both infected and control samples close together. Ultimately it appears that CBA/Ca control and C57BL/6J infected are the two extremes in this PCA with C57BL/6J control resembling CBA/Ca infected more closely than it does CBA/Ca control.

**Fig. 2:**
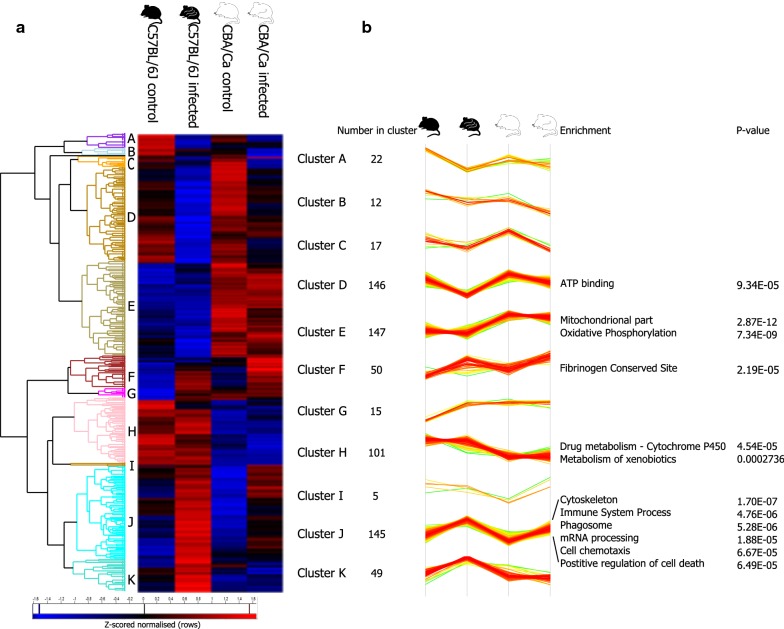
Two-way unsupervised hierarchical clustering of the median protein expression values of all statistically significant differentially abundant proteins. **a** Hierarchical clustering (columns) showed 11 (A–K) distinct clusters. **b** A more detailed view of the clusters with the number of proteins in each cluster, enrichments with associated *P*-values are shown where applicable. If no enrichments are outlined, this indicates no statistically significant enrichments were found. The line graph shows the profiles of the proteins within each cluster, where every line is a protein

Using hierarchical clustering on Z-scored normalised LFQ intensities, a heatmap was produced (Fig. 2a) comprising eleven clusters (A-K) (Additional file 1: Tables S1–S3). Each cluster represents proteins that are expressed in the same pattern in our four groups (Fig. 2b). Within each cluster, there are proteins that are statistically significantly (ANOVA, Ben-Ho FDR < 0.05) expressed (Fig. 2b). The proteins within each cluster were interrogated for GO and KEGG term statistical enrichment (Fisher’s exact test with Ben-Ho adjustment, *P* < 0.05) in Perseus and a number of clear trends became evident. CBA/Ca mice both with and without infection had higher abundances of proteins involved in oxidative phosphorylation (Ben-Ho FDR < 0.0001) and associated mitochondrial proteins (Ben-Ho FDR < 0.0001) (Cluster E). Conversely, C57BL/6J mice both with and without infection had higher abundances of proteins involved in drug metabolism (cytochrome P450) (Ben-Ho FDR < 0.01) and the metabolism of xenobiotics (Ben-Ho FDR < 0.05) (Cluster H). Immune system process (*P* < 0.0001), phagosome (Ben-Ho FDR < 0.0001), cytoskeleton (*P* < 0.0001), mRNA processing (Ben-Ho FDR < 0.01), cell chemotaxis (Ben-Ho FDR < 0.01) and positive regulation of cell death (Ben-Ho FDR < 0.01) are all enriched under infection for both strains.

In total, 2065 proteins were identified (Additional file 2: Table S4). Two-sample t-tests were performed between strains and within strains using an FDR of 0.05 and S_0_ = 0.1 in Perseus software (Table 1). The proteins that were found to be statistically significant were analysed using STRING, Perseus and KEGG.

**Table 1:** Pathways found to be enriched in two-sample t-tests. The numbers of proteins in each t-test are shown below

Pathway	Control		Infected		C57		CBA		Total no. of proteins
	CBA	C57	CBA	C57	Control	Infected	Control	Infected	
Mitochondrial part	34	8	63	24	25	25	2	5	326
Oxidative phosphorylation	11	2	23	5	4	4	0	1	74
Retinol metabolism	3	7	4	10	4	5	0	0	26
Immune system process	7	10	19	26	5	41	1	25	116
Innate immune response	4	1	9	7	2	17	1	12	39
Complement and coagulation cascade	2	1	9	4	1	11	0	6	27
Peroxisome (GOCC)	10	4	22	9	13	7	1	1	60
Ribosome	0	0	0	1	1	3	7	0	70
Cell death	3	5	8	10	3	14	0	7	71
Cytochrome P450	4	5	5	9	7	4	1	0	28

### CBA/Ca control compared to C57BL/6J control

**Fig. 3:**
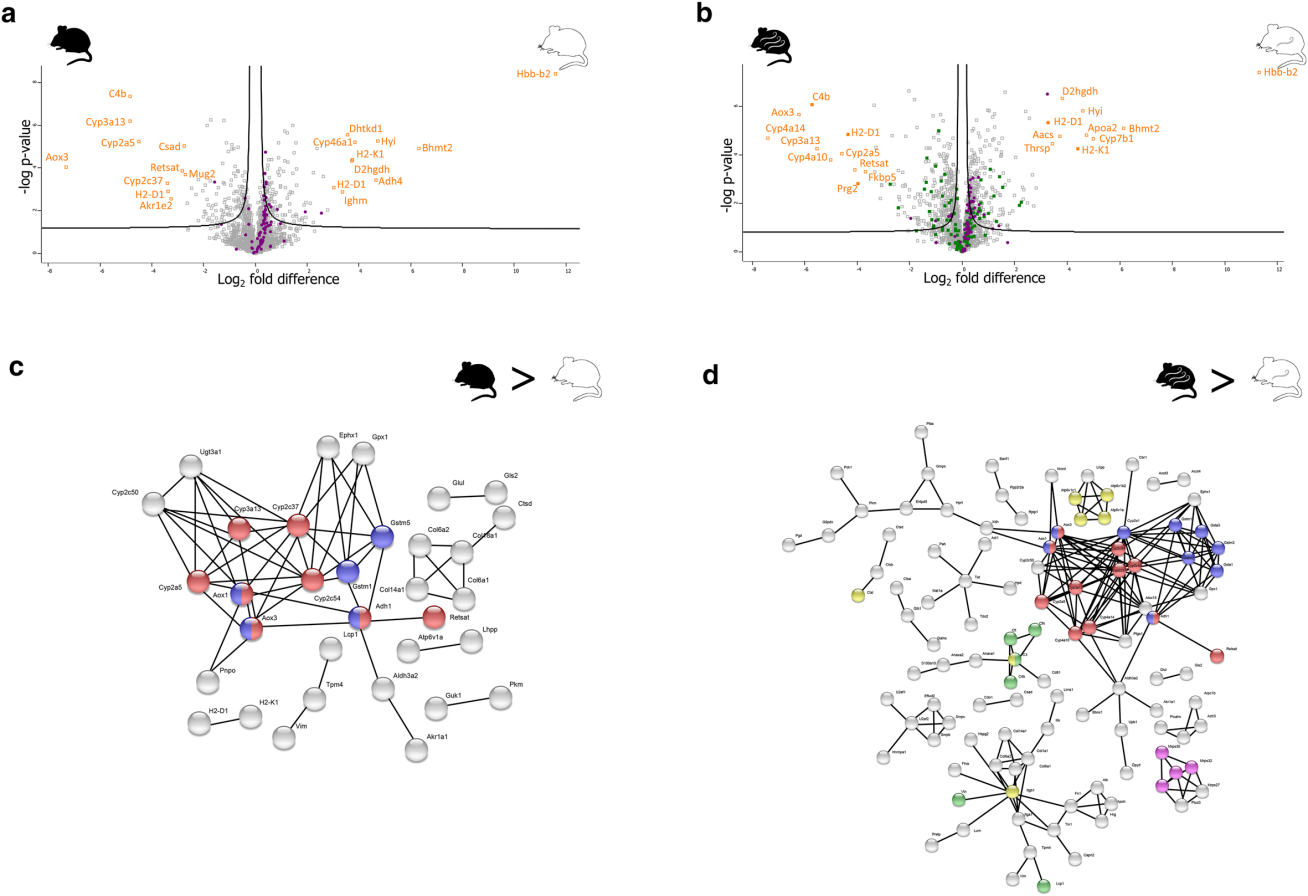
Between strain analysis of abundant proteins. **a** Volcano plot of control mice. On the X-axis are the log_2_ fold differences and on the Y-axis is the − log(P) value. The black curve indicates the cutoff for statistically significant proteins with FDR of 0.05 and S_0_ = 0.1. The top 20 most abundant proteins are highlighted in orange. Proteins involved in the oxidative phosphorylation are highlighted in purple. **b** Volcano plot of infected mice. On the X-axis are the log_2_ fold differences and on the Y-axis is the − log(P) value. The black curve indicates the cutoff for statistically significant proteins with FDR of 0.05 and S_0_ = 0.1. The top 20 most abundant proteins are highlighted in orange. Proteins involved in the oxidative phosphorylation are highlighted in purple. Immune system proteins are highlighted in green. **c** String network analysis of differentially abundant proteins upregulated in C57BL/6J control mice when compared to CBA/Ca control mice, indicated as C57BL/6J>CBA/Ca. Proteins marked in red are part of retinol metabolism. Blue marked proteins are part of the cytochrome P450. If proteins are involved in both pathways, their representative dots are coloured half and half. **d** String network analysis of C57BL/6J infected mice when compared to CBA/Ca infected mice. Red proteins are part of retinol metabolism. Blue marked proteins are part of the cytochrome P450 proteins. Proteins in purple are part of the mitochondrial small ribosomal subunit. Immune system proteins are green and phagosome proteins are yellow

In total, 236 proteins were found to be statistically significant with a log_2_ fold change ranging from − 7.3 to 11.5 (Additional file 2: Table S5). The top 20 most differentially abundant proteins can be seen on the volcano plot (Fig. 3a).

The three most abundant proteins for CBA/Ca were: haemoglobin subunit beta-2, S-methylmethionine-homocysteine S-methyltransferase BHMT2 and putative hydroxypyruvate isomerase. The protein haemoglobin subunit beta-2 had a log_2_ fold change of 11.5, the highest difference in the dataset. Functional enrichment in Perseus revealed that mitochondrial proteins, more precisely, proteins involved in OXPHOS, were more abundant in the CBA/Ca strain. This was confirmed using STRING analysis (KEGG pathway ID: 00190, FDR < 0.0001).

For C57BL/6J the three most abundant proteins were: aldehyde oxidase 3, cytochrome P450 3A13 and complement C4-B. Clusters involved in retinol metabolism (KEGG pathway ID 00830, FDR < 0.0001) and drug metabolism: cytochrome P450 (KEGG pathway ID 00982, FDR < 0.0001) were also identified (Fig. 3c).

### CBA/Ca infected compared to C57BL/6J infected

This pairwise comparison identified 580 statically significant proteins with a log_2_ fold change range from -7.4 to 11.3 (Additional file 2: Table S6). The 20 most SSDA proteins can be found in Fig. 3b. The three most abundant proteins for CBA/Ca were: haemoglobin subunit beta-2, S-methylmethionine-homocysteine S-methyltransferase BHMT2, and 25-hydroxycholesterol 7-alpha-hydroxylase (Fig. 3d). The CBA/Ca strain showed a higher abundance of mitochondrial proteins, in particular, proteins involved in the OXPHOS (KEGG pathway ID: 00190, FDR < 0.0001). CBA/Ca also have more differentially abundant proteins involved in the immune response, more precisely the membrane attack complex (MAC) (cellular component GO ID: 0005579, FDR < 0.0001).

As for the C57BL/6J strain, the three most abundantly expressed proteins were: cytochrome P450 4A14, aldehyde oxidase 3 and complement C4-B. These C57BL/6J mice have more differentially abundant proteins involved in cytochrome P450 complex (Fig. 3d). C57BL/6J showed upregulation in cell-junction proteins (Cellular Component GO pathway ID: 0030054, FDR < 0.0001) and lysosome in (KEGG pathway ID: 04142, FDR < 0.0001). One cluster in specific includes the proton-transporting V-type ATPase complex (cellular component GO pathway ID: 003176, FDR < 0.0001). A cluster in glutathione metabolism (KEGG pathway ID: 00480, FDR < 0.0001) and a small cluster of the small subunit of mitochondrial ribosomal proteins (cellular component GO pathway ID: 0005763, FDR: 0.00746) were also identified. Finally, retinol metabolism is also upregulated compared to CBA/Ca (KEGG pathway ID: 00830, FDR < 0.0001).

### C57BL/6J infected compared to C57BL/6J control

**Fig. 4:**
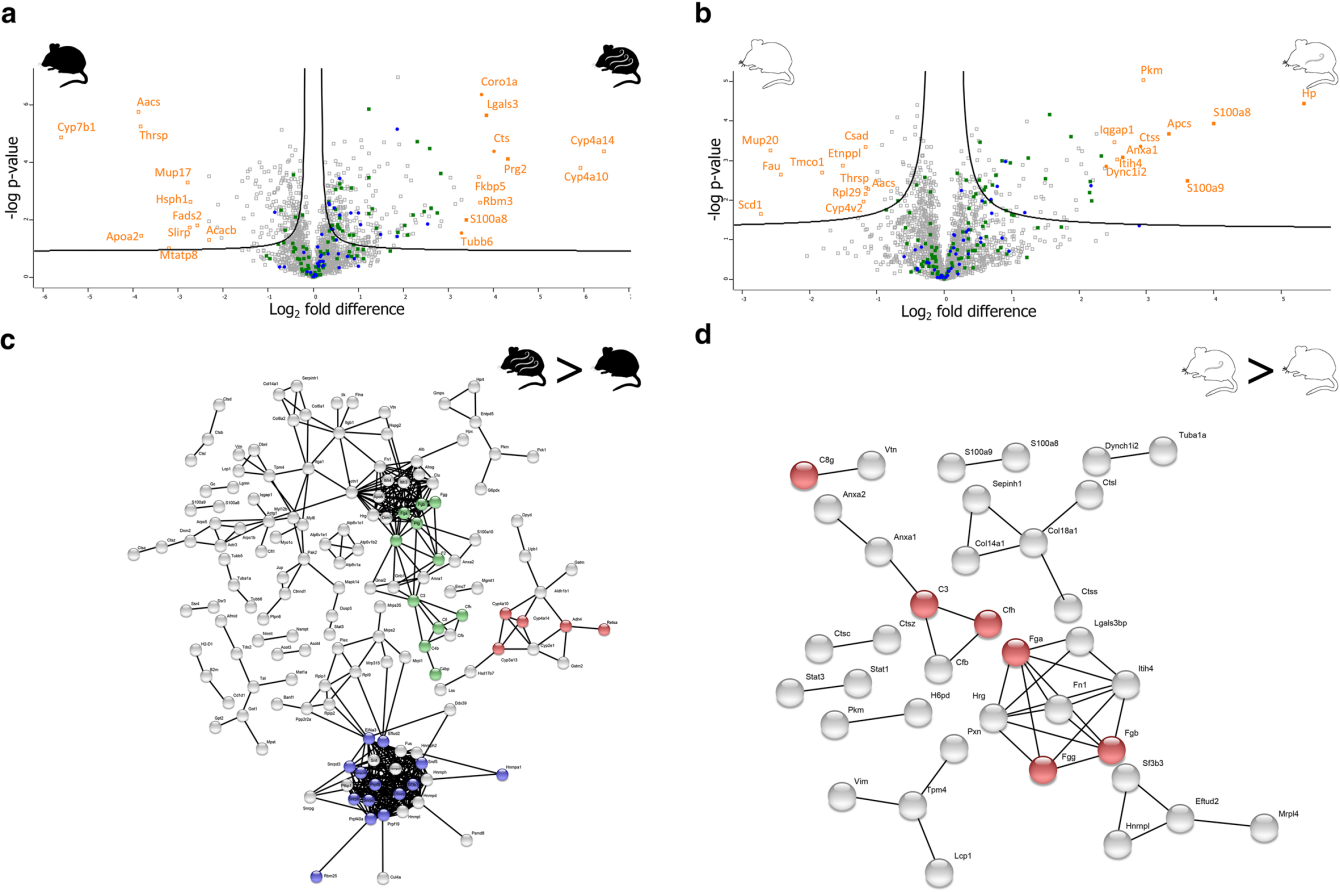
**a** C57BL/6J infected compared to C57BL/6J control. Volcano plot showing the differentially abundant proteins of C57BL/6J mice. On the X-axis are the log_2_ fold differences and on the Y-axis is the − log(P) value. The black curve indicates the cutoff for statistically significant proteins with FDR of 0.05 and S_0_ = 0.1. Proteins abundant in control mice are on the left of the plot and abundant proteins present in infected mice are on the right of the plot. The top 20 most abundant proteins are highlighted in orange. Immune proteins are green squares and proteins involved in the phagosome are blue circles. **b** Volcano polt of CBA/Ca infected compared to CBA/Ca control. On the X-axis are the log_2_ fold differences and on the Y-axis is the − log(P) value. The black curve indicates the cutoff for statistically significant proteins with FDR of 0.05 and S_0_ = 0.1. Proteins abundant in control mice ae on the left of the plot and abundant proteins present in infected mice are on the right of the plot. The top 20 most abundant proteins are highlighted orange. Immune proteins are green squares and proteins involved in the phagosome are blue circles. **c** C57BL/6J infected compared to C57BL/6J control. String network analysis of the proteins found to be differentially abundant in C57BL/6J infected mice when compared to C57BL/6J controls. Red is retinol metabolism. Blue is spliceosome proteins and green is complement pathway. **d** CBA/Ca infected compared to CBA/Ca control. Visualisation of a STRING network analysis performed on the differentially abundant proteins of CBA/Ca in infected mice when compared to CBA/Ca control mice. Proteins highlighted in red are the complement pathway

In total 451 proteins were statistically significant for this two sample t-test, (Additional file 2: Table S7) with a log_2_ fold change range from − 5.5 to 6.4 (Fig. 4a). For the control samples, the three most abundantly expressed proteins were: 25-hydroxycholesterol 7-alpha-hydroxylase, acetoacetyl-CoA synthetase, and thyroid hormone-inducible hepatic protein. Functional enrichment revealed a trend towards translational upregulation; however, most proteins were under the FDR cutoff. A similar situation was seen for proteins involved in oxidative phosphorylation and peroxisomal proteins. STRING analysis confirmed the increased abundance of mitochondrial proteins (cellular component GO ID: 0005739, FDR < 0.0001) and proteins involved in the metabolism of xenobiotics by cytochrome P450 (KEGG pathway ID: 00980, FDR < 0.0001). Additionally, retinol metabolism proteins were found to be upregulated (KEGG pathway ID: 00830, FDR < 0.0001).

For the infected samples, the most abundantly expressed proteins were: cytochrome P450 4A14, cytochrome P450 4A10 and eosinophil granule major basic protein. An increase in RNA and DNA binding proteins and proteins involved in RNA processing under infection was identified (biological process GO: 0006396, FDR < 0.0001). A higher abundance of proteins involved in the regulation of cell death and immune system process in the infected samples was also identified, including immune system process (biological process GO: 0002376, FDR < 0.0001) and more specifically the complement and coagulation cascade (KEGG pathway ID: 04610, FDR < 0.0001) (Fig. 4c). Finally, an increase in phagosomal proteins under infection was also found (KEGG pathway ID: 04145, FDR < 0.0001).

### CBA/Ca infected compared to CBA/Ca control

In this two sample t-test, 117 proteins were statistically significant (Additional file 2: Table S8), with a log_2_ fold change range from − 2.7 to 5.3. The most abundantly expressed proteins for the control samples were: acyl-CoA desaturase 1, major urinary protein 20, and 40S ribosomal protein S30 (Fig. 4b). Functional enrichment of the control samples revealed an upregulation of ribosomal proteins and the translational machinery in control samples analysis (KEGG pathway ID: 03010, FDR: 0.0031).

The most abundantly expressed proteins for the infected samples were: haptoglobin, protein S100-A8 and protein S100-A9, proteins involved in transcription those of the innate immune system in particular. The proteins of the immune response (biological process GO: 0006955, FDR < 0.0001) were upregulated in infected samples compared to control samples (Fig. 4d). Phagosome proteins were found to be upregulated (KEGG pathway ID: 04145, FDR < 0.0001). Additionally, the analysis revealed RNA binding proteins to be upregulated in the infected group (molecular function GO ID: 0003723, FDR < 0.0001). Regulation of cell death was found to be upregulated, and although no clear cluster existed in STRING analysis the regulation of cell death process (biological process GO: 0010941, FDR < 0.0001) was found to be enriched. Additionally, changes in cell movement were observed including upregulation of proteins involved in cytoskeleton organisation, cell migration and cell-cell adhesion.

### Enriched pathways

#### The susceptible strain (C57BL/6J) has more immune system process proteins

A total of 116 proteins were identified from the immune system process, including 39 from the innate immune response and 27 from the complement and coagulation cascade (Table 1). Under infection, C57BL/6J mice produce more immune response-associated proteins (26 proteins) than their CBA/Ca counterparts (19 proteins). C57BL/6J infected mice also showed a higher abundance of immune system process proteins (41 proteins) when compared to their controls. CBA/Ca infected mice also have a higher abundance for proteins associated with immune system process (25 proteins) compared to their own controls.

Complement factor i (Cfi) and h (Cfh) were upregulated in C57BL/6J infected mice compared to CBA/Ca infected mice. These proteins are involved in inhibiting the complement response. In contrast, mannose-binding protein A (Mbl1) and mannose-binding protein C (Mbl2), both proteins essential for activation of the lectin complement pathway, are found in CBA/Ca infected mice when compared to C57BL/6J infected mice. The same is true for part of the membrane attack complex (MAC), complement component C8 gamma chain (C8g), C8b, C8a and C9 and tapasin (Tapbp).

When comparing C57BL/6J infected mice with their own controls, more immune system-associated proteins are found to be upregulated under infection: Cfb, involved in alternative complement cascade; and C4bp, involved in controlling the classical complement cascade. Additionally, factors involved in blood clotting are upregulated [plasminogen, fibrinogen gamma chain (Fgg), Fga, Fgb, C3 and tyrosine-protein phosphatase non-receptor type 6] and platelet glycoprotein 4 (CD36).

As for CBA/Ca, Cfb, a protein of the alternative pathway, is upregulated under infection when compared to its counterpart control, together with C8g, C3 and Cfh. The latter is an inhibitor of the complement cascade.

#### Mitochondrial proteins are upregulated in the resistant (CBA/Ca) strain

Of the 2065 identified proteins, 326 were found to be associated with the mitochondria, with 204 belonging to the mitochondrial membrane and 74 involved in oxidative phosphorylation. Increased mitochondrial protein abundance was observed in the CBA/Ca strain in comparison to the C57BL/6J strain, both intrinsically and under infection (Table 1). Furthermore, no change in absolute protein numbers was observed when comparing the C57BL/6J group between its controls and infected. For the CBA/Ca strain, however, a small increase was observed.

#### Differences in retinol metabolism between the two mouse strains

In total, 26 of the 2065 proteins identified are involved in retinol metabolism (Table 1). The C57BL/6J strain showed a higher abundance of these proteins when compared to the CBA/Ca mice both with and without infection. When comparing infected livers of the CBA/Ca strain with corresponding controls, no difference in retinol metabolism was observed. The C57BL/6J mice, however, express different proteins between control and infection. The protein all-trans-retinol 13,14-reductase (Retsat) is among the 10 most abundant proteins in C57BL/6J control when compared to CBA/Ca control.

#### Major urinary proteins

Major urinary proteins (Mup) are downregulated under infection. Mup17 is present in the top 10 most differentially upregulated proteins in the control group of the C57BL/6J when compared to its infected counterparts. The same is true for Mup 20 in the CBA/Ca group. In total, 6 Mups are upregulated in C57BL/6J controls when compared to C57BL/6J infected. As for the CBA/Ca group, 2 Mups are found to be upregulated in controls when compared to the infected group. When comparing both control samples, Mup20 is found to be upregulated in C57BL/6J mice. This same protein remains more abundantly expressed in C57BL/6J infected samples, when compared to CBA/Ca infected mice. However, CBA/Ca infected mice also showed an increase in Mup17, Mup1, Mup3 and Mup2 when compared to C57BL/6J infected mice. Our dataset shows a downregulation of Mup20 and Mup2 under infection for CBA/Ca when compared to their own controls. As for C57BL/6J mice, Mup6, Mup20, Mup2, Mup3, Mup1 and Mup17 are downregulated under infection when compared to their own controls. In essence, the C57BL/6J strain downregulates more Mups (6) under infections when compared to its own control than the CBA/Ca strain (2).

#### S100

S100a8 and S100a9, two components of the heterodimer calprotectin, were found to be highly abundant in this dataset. S100a8 and S100a9 are in the top 3 most abundant proteins in CBA/Ca infected when compared to its control counterparts. For C57BL/6J infected mice, S100a8, S100a9, S100a10 and S100a1 are differentially expressed compared to their controls.

To conclude, CBA/Ca mice show an increase in complement activating proteins under infection, whereas the C57BL/6J infected mice have a higher abundance of complement inhibiting proteins. Additionally, CBA/Ca mice have a higher abundance of OXPHOS proteins compared to the C57BL/6J, both intrinsically and under infection. Furthermore, a difference was observed in the retinol metabolism proteins, where the C57BL/6J strain shows a higher abundance in these proteins than the CBA/Ca strain.

## Discussion

Ascariasis affects 800 million people worldwide [1] and, although anthelmintic drugs are available for its treatment, reinfections occur frequently, due principally to the persistence of infective eggs in the environment. Despite the extensive number of infected people, our knowledge of the molecular mechanisms involved in larval migration and the host immunological response remains incomplete. The role of the liver in ascariasis has been understudied, yet this appears to be the primary location where the difference in resistance and susceptibility manifests. This study extends our previous work that focused on the liver at day 4 p.i. [19], by examining the liver at a later time point, day 7 p.i. At day 4 p.i., we identified molecular determinants that were possibly associated with resistance whereas in this study, with a focus on day 7 p.i., we attempt to obtain insight into the responses to, and effects of *Ascaris* infection at the molecular and immunological level at a time where the biggest difference in larval burden between the CBA/Ca and C57BL/6J mouse strains is observed.

Four different groups of proteins were identified including those associated with mitochondrial processes, the immune response, retinol metabolism and haemoglobin, all of which showed both an intrinsic difference between the two strains as well as a difference between control and infected groups.

### The immune response to the parasite differs between the two strains

In comparison to the responses previously reported for day 4 p.i., a more pronounced innate immune response is observed at day 7 p.i. with differences observed between the C57BL/6J and CBA/Ca strains. The C57BL/6J mouse strain had a higher abundance of immune system process proteins under infection. However, this does not necessarily translate into a higher activation of the immune system. A more thorough investigation of exactly which type of proteins are involved reveals a difference in innate immune response activity.

In particular, we detected a difference in the complement system, with a higher abundance of activating and inactivating proteins in the CBA/Ca and C57BL/6J strains, respectively. The CBA/Ca strain displayed an increased abundance in proteins involved in the lectin pathway of the complement system, more specifically, with both Mbl1 and Mbl2 being statistically significantly upregulated in CBA/Ca infected when compared to C57BL/6J infected. Conversely, C57BL/6J infected had more proteins upregulated involved with complement inhibition, Cfi and Cfh in particular, when compared to its CBA/Ca infected counterpart. Additionally, Cfb was upregulated in C57BL/6J infected mice compared to their uninfected controls. This suggests a downregulation of complement activation in the C57BL/6J strain and an upregulation of the lectin pathway in the CBA/Ca strain. So we observed a different activation of the immune system, with the C57BL/6J strain actually downregulating the immune response. It is unclear whether this is a response from the host or immune modulation by the parasite.

Complement is one of the first steps in the innate immune system and plays an important role not only in directly killing a pathogen (MAC) but also in the activation of various other members of the immune system [33]. It is especially a powerful mechanism that provides a link between the innate and adaptive immune response [34]. Complement is thus an important first step in the activation of the immune response against invading pathogens. Unsurprisingly, some parasites are capable of inhibiting this mechanism, such as *Brugia malayi* which has a serine protease that inactivates C5a, a key component in the cascade [35]. Early granulocyte-mediated larval attrition of *Nippostrongylus brasiliensis* appears to be in part complement-driven as well, with the alternative pathway being activated during early invasion and a switch to the lectin pathway during late infection [36]. Similarly, *Onchocerca volvulus* microfilariae have been shown to bind factor H, which can cleave C3b to iC3b in the presence of factor I, therefore inactivating the central C3b protein [37]. This resistance to complement activation throughout maturation, with a switch to a different mechanism, has also been seen in *O. volvulus* and *Dirofilaria immitis* as the parasites mature into the next life cycle stages [38, 39]. Recombinant *Trichinella spiralis* paramyosin can bind C8 and C9 and therefore inhibit MAC formation [40]. More specifically, Mbl has been found to bind oligosaccharides on the surface of *T. spiralis* larvae [41]. Additionally, Mbl-A was found to bind to the surface of *B. malayi* and activate C3 [42].

In short, a range of parasites have developed mechanisms to inhibit or modify complement activation. Little evidence is available for the role of complement in *Ascaris* infection. However, one *in vivo* study stimulated alveolar macrophages from male Wistar rats with somatic antigens from *A. suum* adult worms and L3 stage larvae, produced nitric oxide (NO) in a dose-dependent manner [43]. As for mouse studies, C3 from both human and guinea pig serum has been shown to bind *A. suum* larvae [44]. There are no uniform specialized pattern recognition receptors (PRRs) for helminths or *Ascaris* [45] which could be recognized by the complement system; this is in stark contrast with the highly specialized PRRs for viruses and bacteria. However, the presence of C-TL like protein on *A. suum* [46] could indicate a role in immune evasion or tissue recognition [45].

As described above, complement modulatory mechanisms have been observed for several parasites and it may be that *Ascaris* either directly or indirectly modulates host immune components during infection also. Components of adult *A. suum* body extract and fluid have both been shown have a strong immune modulatory effect inhibiting DC maturation, cytokine production and results in the alteration of human macrophage surface markers [46–48]. It appears that the ability of *Ascaris* to modulate the host response is particularly mouse strain dependant. However, the mechanisms of how *Ascaris* evades or suppresses the immune system and the factors involved in immunomodulation have yet to be determined.

### Intrinsic, between-strain differences in oxidative phosphorylation

Proteins of OXPHOS were found to be upregulated in the CBA/Ca strain, both with and without infection, when compared to the C57BL/6J strain. The OXPHOS proteins are part of the mitochondria, which function as the cells’ powerhouse, by producing adenosine triphosphate (ATP) at the end product of the electron transport chain (ETC) [49]. As reactive oxygen species (ROS) are produced during OXPHOS we speculate that the CBA/Ca strain, therefore, could have higher ROS concentrations. This higher ROS concentration could be of great benefit in order to ward off the invading parasite. ROS could directly damage the parasite but it is also known to act as a signalling molecule, including in the activation of the immune system. Mitochondrial ROS (mROS) specifically has been shown to be able to act as a danger-associated molecular pattern (DAMP) [49] and is known to direct the immune system by polarizing macrophages and differentiating CD4^+^ T cells during infection [50].

The mitochondrial role in parasite immunology is as yet poorly characterised with only a small number of studies performed. In *Toxoplasma gondii* the parasitic vacuole binds with the host mitochondrial proteins [51, 52]. This is mediated by the parasites’ secreted mitochondrial association factor 1 (MAF1). Certain *T. gondii* strains are able to bind mitochondrial proteins, this could indicate that the different strains have altered susceptibility to ROS [52]. In general, uncoupling proteins are involved in reducing the production of mROS by decreasing the mitochondrial membrane potential [53]. In another example, livers of Wistar rats infected with the parasitic helminth *Cysticercus fasciolaris* showed an increase of ROS compared to their uninfected counterparts [54].

In order to maintain a normal redox status within the parasite and to contain ROS attacks from hosts, the parasite needs a good detoxification strategy. *A. suum* adult worms are known to have peroxiredoxin [55], glutathione S-transferase (GST) [56] and catalase [57]. Additionally, peroxiredoxin was found to be present in an L3 stage larvae cDNA library of *A. suum*, indicating the importance of this protein as a ROS defense mechanism [55]. GST was found in the excretory/secretory products of *A. suum* L4 lung stage larvae [58]. Catalase activity was found to be highest in the unembryonated *A. suum* eggs and decreased steadily during embryonation [59]. The catalase activity during the L3 life stage was found to be similar to that of the adult life stages. To summarise, *A. suum* larvae have been shown to possess antioxidant activity which could be used as a defense mechanisms against ROS attacks from the host.

### Retinol pathways involving cytochrome P450 are more pronounced in the susceptible (C57BL/6J) strain

The appearance of proteins involved in retinol metabolism was of particular interest. The names retinol and vitamin A are sometimes used interchangeably, but in general vitamin A is a broader term which includes all ‘compounds having the biological activity of retinol or its metabolic products’ [60]. The vitamin A status of people infected with soil transmitted helminths (STH), *Ascaris* in particular, has been studied extensively, with some studies indicating that vitamin A supplementation reduced reinfection rates [61, 62]. However, one randomised controlled trial in a high prevalence STH endemic area dispute this claim [63].

In our study, we found that C57BL/6J have a higher abundance of proteins involved in retinol metabolism when compared to CBA/Ca, both with and without infection. Additionally, C57BL/6J infected mice have a higher abundance of these proteins when compared to their controls. As for CBA/Ca, no difference in the abundance of proteins involved in the retinol metabolism was found between infected and uninfected controls.

We found more proteins involved in retinol metabolism in C57BL/6J control mice compared with CBA/Ca mice. In general, more proteins are involved with the formation and degradation of all-trans-retinoate or retinoic acid (atRA) in the C57BL/6J strain when compared to its CBA/Ca controls.

This difference remains true when comparing both strains under infection. In particular, the cytochrome P450 Cyp3A13 is the fourth-most abundant protein in this comparison. Other studies have identified a change in abundance in this protein, with hepatic Cyp3A being downregulated in CBA/Ca mice infected with *T. gondii* [64]. This is in contrast with our own findings where the CBA/Ca mice showed an upregulation of proteins involved in retinol-retinal conversion. In another study, male ICR mice infected with *Babesia microti* showed a similar reduction in hepatic CYP3A at 12 days p.i. [65].

Retinoic acid (atRA) is transcriptionally active and has a wide range of functions through binding with the retinoic acid receptors (RAR) and retinoid X receptors [66]. In the C57BL/6J mice under infection, when compared to CBA/Ca infected mice, we observed a higher abundance of Cyp3A and Cyp1A proteins. These proteins degrade the atRA and therefore could play a role in reducing its transcriptional function [67]. atRA has a wide range of functions including immunity, vision and reproduction [68]. The influence of atRA on the immune system during parasitic infection is variable [69]. Vitamin A insufficient mice orally infected with *T. gondii* showed impaired Th1 and Th17 response on the mucosal level [70]. A similar outcome was seen in Rara^−/−^ mice, indicating that the atRA/RAR is essential for T-cell priming [70].

Some data regarding the importance of atRA in *Ascaris* infection is already available. Pigs fed atRA showed, an increased abundance of hepatic mRNA of Th2-associated cytokines; this was true for both pigs infected with *A. suum* and their uninfected controls [71]. The presence of atRA might, therefore, indicate an early push for a Th2 shift. This could be what is absent in the C57BL/6J mice if atRA is being degraded by Cyp3A proteins. Interestingly, ABA-1, an allergen [72, 73] found in somatic tissues of *Ascaris* and secreted by the parasite [74, 75], has been found to bind retinol among other substances such as fatty acids [76]. This could indicate a regulatory mechanism of the parasite on retinol levels in the host and therefore control the Th2 response mounted by the host [66].

In short, it is unclear what the exact role of the retinol metabolism is in this mouse model of *A. suum* infection. However, based on previous findings in human populations it appears that there might be an important role for vitamin A, its storage and metabolism. Additionally, there is a link between Retsat protein, which is one of the ten most abundant proteins in C57BL/6J control when compared to CBA/Ca control, and oxidative stress [77]. Retsat is an enzyme involved in retinol metabolism. Its role in ROS production is currently unclear, but the enzyme could be a potential link between retinol metabolism and the immune system.

### Other proteins that are not part of the above processes

#### Major urinary proteins

At day 7 p.i. we found that in general Mups were downregulated under infection for both strains when compared to their respective controls. A total of six Mups were observed in the dataset. Mups are pheromone transporters and are used as chemical cues for individual recognition [78–80]. They are produced in the liver, under the regulation of testosterone and subsequently released in the plasma, from where they are excreted through the urine [81]. Mups bind small volatiles [81], a process that is necessary to ensure their slow evaporation once released through urine [82]. Both males and females produce Mups. However, male adult mice produce more Mups than females [83]. A decrease in Mups in mice livers is also observed during *S. mansoni* infection [80, 84] highlighting that a potentially conserved response to parasitic infection may be present and localised to the liver.

#### S100

In our dataset, the proteins S100a10, S100a9, S100a8 and S100a1 were all upregulated in C57BL/6J infected compared to its own control. Only s100a8 and s100a9 are upregulated in CBA/Ca infected compared to their own control. In short, S100a10 and S100a1 are only significantly upregulated in C57BL/6J. These proteins were absent in the dataset of the day 4 p.i. proteomes.

The S100 group is a large group of proteins involved in a number of cellular functions including cell motility and cell differentiation [85]. The S100a8 and S100a9 proteins can be found as homodimers, but can also form a heterodimer, called calprotectin, when in the presence of calcium. Calprotectin can act as a DAMP [86, 87] and it can also recruit neutrophils to the site of infection. Such is the case in *S. japonicum* infection, where S100a8 and neutrophils were found near fibrotic areas of granulomas [88]. During *Litomosoides sigmodontis* infection, BALB/c mice were found to have an increase in S100a9 transcription in lung tissue within hours after the infection [89]. The authors believe the role of s100a9 in filarial infection is either as a danger signal or as part of neutrophil extracellular traps (NETs) [90] NETs have been found to play a role in the clearance of pathogens, such as trapping *Leishmania amazonensis in vivo* [91] and activating TLR 2 and 4 during *Trypanosoma cruzi* infection [92].

Regardless of whether the S100a8 and S100a9 proteins are DAMPs or recruit neutrophils, they appear to play an important role in leucocyte (neutrophilic) defence against the invading larvae.

## Conclusions

These results confirm our previous findings where we identified an innate difference between the two mouse strains with the CBA/Ca strain showing a higher abundance of OXPHOS proteins both with and without infection. Here, we focused on a later time point and were able to identify novel hepatic responses to the invading parasite. We found that the initiation of the complement system of the innate immune response differs between the two strains. We distinguished an increased abundance of proteins involved in the activation of the complement system *via* the lectin pathway in the CBA/Ca strain. This was in contrast to the C57BL/6J strain which showed a higher abundance of proteins involved in inhibition of this cascade. This difference in immune activation is an interesting new finding with potentially important consequences for the overall immune response towards the parasite. Furthermore, our finding regarding the retinol pathway is remarkable given the many human studies that have been performed on vitamin A supplementation and STH (re)infection. Our results confirm a potential role for retinol and its metabolites during *Ascaris* infection in the liver. However, it is unclear what this role is and how retinol metabolism influences the hosts’ response. Further studies will be essential to unravel these questions. In short, this study confirms that both mouse strains show a different hepatic proteomic response to *A. suum* infection which could give more insight into the predisposition observed in both pigs and humans.

## Additional files

**Additional file 1: Table S1.** ANOVA statistics (*P*- and q-values) for all proteins (Benjamini-Hochberg FDR < 0.05. **Table S2.** All proteins involved in the assembly of the heatmap. **Table S3.** Enriched GO, KEGG and protein family terms from heatmap clusters.

**Additional file 2: Table S4.** Proteins identified from the liver left lobes of C57BL/6J and CBA/Ca mice with and without *Ascaris* infection. **Table S5.** t-test (*P* < 0.05) results and relative fold differences between CBA/Ca control and C57BL/6J control mice. **Table S6.** t-test (*P* < 0.05) results and relative fold differences between CBA/Ca infected and C57BL/6J infected mice. **Table S7.** t-test (*P* < 0.05) results and relative fold differences between C57BL/6J control and infected mice. **Table S8.** t-test (*P* < 0.05) results and relative fold differences between CBA/Ca control and infected mice.

## Data Availability

The mass spectrometry proteomics data have been deposited to the ProteomeXchange Consortium *via* the PRIDE partner repository with the dataset identifier PXD014508, available at http://www.proteomexchange.org/submission/index.html.

## Abbreviations

ATP: adenosine triphosphat
e atRA: retinoic acid
C8g: complement component C8 gamma chain
Cf: complement factor
DAMP: danger-associated molecular pattern
ETC: electron transport chain
FDR: false discovery rate
Fgg: fibrinogen gamma chain
GO: gene ontology
GST: glutathione S-transferase
KEGG: Kyoto Encyclopaedia of Genes and Genomes
LB: lysis buffer
LFQ intensities: normalised protein intensities
MAC: membrane attack complex
MAF-1: mitochondrial association factor 1
Mbl1: mannose-binding protein A
Mbl2: Mannose-binding protein C
mROS: mitochondrial ROS
Mup: major urinary proteins
NET: neutrophil extracellular trap
NO: nitric oxide
OXPHOS: oxidative phosphorylation
p.i.: post-infection
PCA: principal component analysis
Pfam: protein family
PRR: pattern recognition receptor
RAR: retinoic acid receptors
Retsat: all-trans-retinol 13,14-reductase
ROS: reactive oxygen species
SD: standard deviation
SSDA: statistically significant differentially abundant
STH: soil transmitted helminths
STRING: Search Tool for Retrieval of Interacting Genes/Proteins
Tapbp: tapasin
Th: T helper
